# Differential Expression and Pathway Analysis in Drug-Resistant Triple-Negative Breast Cancer Cell Lines Using RNASeq Analysis

**DOI:** 10.3390/ijms19061810

**Published:** 2018-06-19

**Authors:** Safa Shaheen, Febin Fawaz, Shaheen Shah, Dietrich Büsselberg

**Affiliations:** 1 Genomics Central, Thrissur 680125, India; safashaheenshah@gmail.com (S.S.); fawazfebin@gmail.com (F.F.); shaheen@genomicscentral.com (S.S.); 2 Weill Cornell Medicine, Qatar Foundation-Education City, P.O. Box 24144, Doha, Qatar

**Keywords:** Triple-negative breast cancer, drug resistance, RNASeq, cytokine-cytokine receptor interaction, basal b

## Abstract

Triple-negative breast cancer (TNBC) is among the most notorious types of breast cancer, the treatment of which does not give consistent results due to the absence of the three receptors (estrogen receptor (ER), progesterone receptor (PR), and human epidermal growth factor receptor 2 (HER2) as well as high amount of molecular variability. Drug resistance also contributes to treatment unresponsiveness. We studied differentially expressed genes, their biological roles, as well as pathways from RNA-Seq datasets of two different TNBC drug-resistant cell lines of Basal B subtype SUM159 and MDA-MB-231 treated with drugs JQ1 and Dexamethasone, respectively, to elucidate the mechanism of drug resistance. RNA sequencing(RNA-Seq) data analysis was done using edgeR which is an efficient program for determining the most significant Differentially Expressed Genes (DEGs), Gene Ontology (GO) terms, and Kyoto Encyclopedia of Genes and Genomes (KEGG) pathways. iPathway analysis was further used to obtain validated results using analysis that takes into consideration type, function, and interactions of genes in the pathway. The significant similarities and differences throw light into the molecular heterogeneity of TNBC, giving clues into the aspects that can be focused to overcome drug resistance. From this study, cytokine-cytokine receptor interaction pathway appeared to be a key factor in TNBC drug resistance.

## 1. Introduction

Breast cancer represents one of the most common cancers in women and is a major life-threatening disease. In certain cases, due to the development of drug resistance, treatment becomes unsuccessful [1]. Triple-Negative Breast Cancer (TNBC) accounts for about 15–20 percent of the breast cancers. Drugs normally targeted to the receptors do not apply to TNBC as they lack the three receptors, namely the estrogen receptor (ER), progesterone receptor (PR), and human epidermal growth factor receptor 2 (HER2) [2]. TNBC has fewer treatment options and there is limitation of unavailability of specific effective targeted therapy due to the absence of the three target receptors and this makes TNBC cells unresponsive to the conventional hormonal or trastuzumab-based therapy. This results in the comparatively high mortality rate [3]. Chemotherapy is the most effective treatment known, but overcoming drug resistance is a big challenge. TNBC is also known to be aggressive with high metastasis rates as well as poor prognosis [4]. The high level of molecular heterogeneity also demands changes brought in drug administration, which makes combinatorial drug therapy favorable [4,5]. Therefore, it is essential to identify molecular targets for a more effective therapy. Targeted therapy by developing targeted agents like poly(ADP-ribose) polymerase-1 (PARP-1), epidermal growth factor receptor (EGFR), fibroblast growth factor receptor 2 (FGFR2), vascular endothelial growth factor (VEGF), and the mammalian target of rapamycin (mTOR) in combination with chemotherapy seems promising in treatment of TNBC [3]. Pathways such as NOTCH signaling pathway and 5′ adenosine monophosphate-activated protein kinase (AMPK) signaling pathway are being targeted for overcoming drug resistance in cancer therapy [6,7]. Drugs targeting pathways are being developed such as those targeting the phosphatidylinositol-3-kinase (PI3K)/AKT/mTOR pathway for the treatment of TNBC and shows promise as stated by Gradishar W.J. et al. [8].

The subtypes of TNBC are Basal-like (BL-1, BL-2), immune-modulatory, luminal androgen receptor, mesenchymal, and mesenchymal/stem-like subtype and claudin low subtype [9]. SUM 159 and MDA-MB-231 are widely used TNBC cell lines for in vitro studies and both are of Basal B subtype [10]. The bromodomain inhibitor JQ1isan effectively proven drug for TNBC and is being widely used. Bromodomains recruit proteins of transcription machinery by recognizing acetylated-lysine residues in nucleosomal histones. Bromodomain and extra terminal (BET) inhibitors are able to inhibit several transcription factor expressions and thereby result in growth inhibition in several tumors [11]. Dexamethasone (Dex) is widely used as a pretreatment drug in chemotherapy to prevent severe side effects such as nausea and vomiting as well as to prevent allergic reactions. It is a glucocorticoid (GC) and functions by binding to the glucocorticoid receptor(GR) [12]. Bioinformatics is an emerging field being used to analyze and interpret high-dimensional biological data using advanced computational tools. Next-Generation Sequencing technologies (NGS)-based bioinformatics analytics are designed to convert signals to data, data to interpretable information, and information into actionable knowledge. RNA Sequencing (RNA-Seq) is an efficient tool for sequencing the transcriptome using high throughput sequencing technologies, NGS. It helps to understand various gene expressions at any point in time. For example, it helps to determine how a disease develops, how a drug gets affected, and how gene splicing occurs. When compared to microarrays, RNASeq has several advantages such as high technical reproducibility, low background noise, and large dynamic range [13]. It can also be exploited for the identification of novel transcripts and splicing events since it can be analyzed with or without a reference sequence [14]. The analysis part of RNASeq still remains a challenge with continuous improvements occurring in the design of bioinformatic programs used in the analysis. One of the main applications of RNA-Seq analysis lies in drug research, where appropriate drug targets can be designed after studying the gene expression and pathways in a disease condition [15].

In view of this, the present study aimed at studying the gene expression and pathways in TNBC cell lines SUM159 and MDA-MB-231 that show drug resistance and identify the top differentially expressed ones so that appropriate drug targets can be designed for overcoming the mechanism of resistance.

## 2. Results

At a 24-h time interval of JQ1 treatment on SUM159 and SUM159R cell lines, out of the 14,445 genes differentially expressed, a total of 2682 differentially expressed genes (DEGs) were observed to be significantly differentially expressed between SUM159R and SUM159 (891 upregulated and 1791 downregulated genes) at a *p*-value of 0.05 and log fold change (log FC) of 1. At a 4-h time interval of Dex treatment, out of the 13,280 genes differentially expressed between Dex-treated MDA-MB-231 cell lines and vehicles (control samples), a total of 563 genes were observed to be significantly differentially expressed (325 upregulated and 238 downregulated genes) at a *p*-value of 0.05 and log FC of 1 (Table 1).

The upregulated genes with lowest *p*-values were *SLC37A2* and *CADM1* in SUM159R cells treated with JQ1 at 3 and 24 h, respectively, whereas the downregulated genes with the lowest *p*-values were *DCN* and *CDH11*. In MDA-MB-231 cells treated with Dexamethasone at two and four hours, the upregulated gene with lowest *p*-values was *PDK4* in both time intervals, whereas the downregulated genes with lowest *p*-values were *PLK2* and *PRAG1*. The top most significantly up-and downregulated differentially expressed genes are given in Table 2. The top ten pathways for each sample are given in supplementary material (Supplementary Table S1a–d).

**Figure 1:**
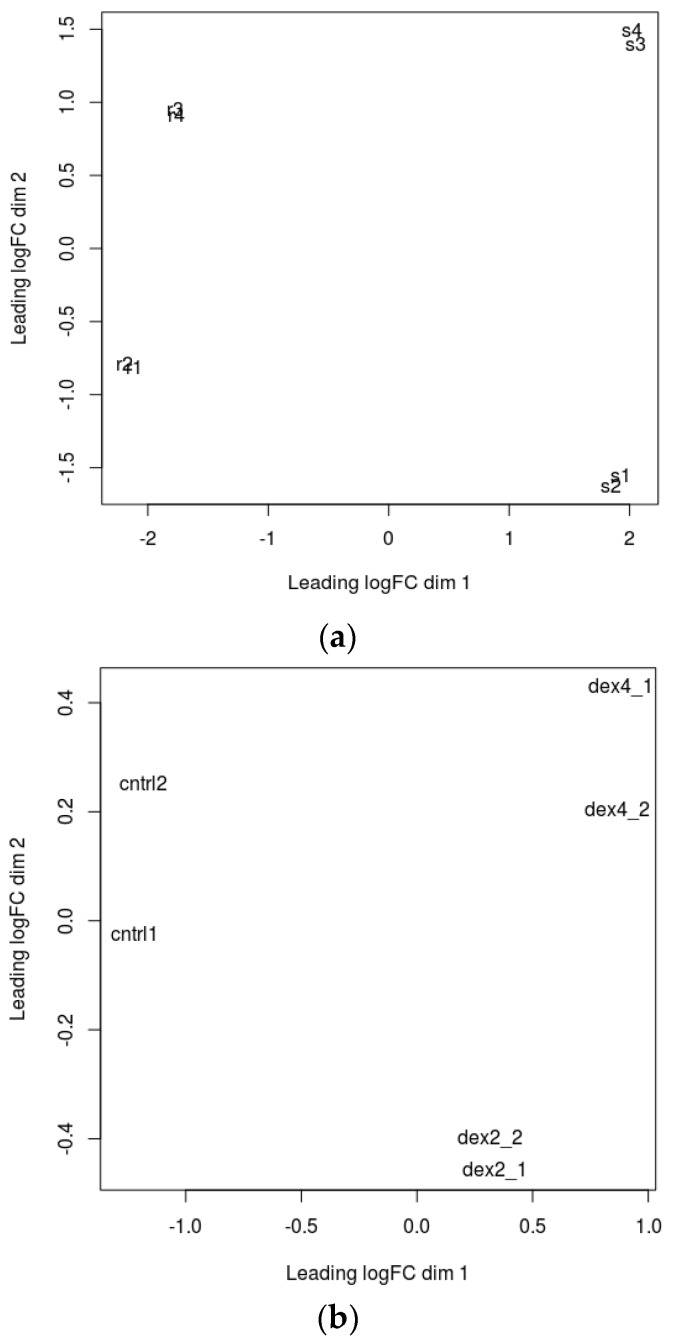
Multidimensional scaling plots (**a**) for JQ1 treatment; (**b**) for Dexamethasone treatment. s1—JQ1 sensitive-3 h treatment, s2—JQ1 sensitive-3 h treatment, s3—JQ1 sensitive-24 h treatment, s4—JQ1 sensitive-24 h treatment, r1—JQ1 resistant-3 h treatment, r2—JQ1 resistant-3 h treatment, r3—JQ1 resistant-24 h treatment, r4—JQ1 resistant-24 h treatment, ctrl1—control 1, ctrl2—control 2, dex2_1—Dexamethasone-2 h treatment, dex2_2—Dexamethasone-2 h treatment, dex4_1—Dexamethasone-4 h treatment and dex4_2—Dexamethasone-4 h treatment.

**Figure 2:**
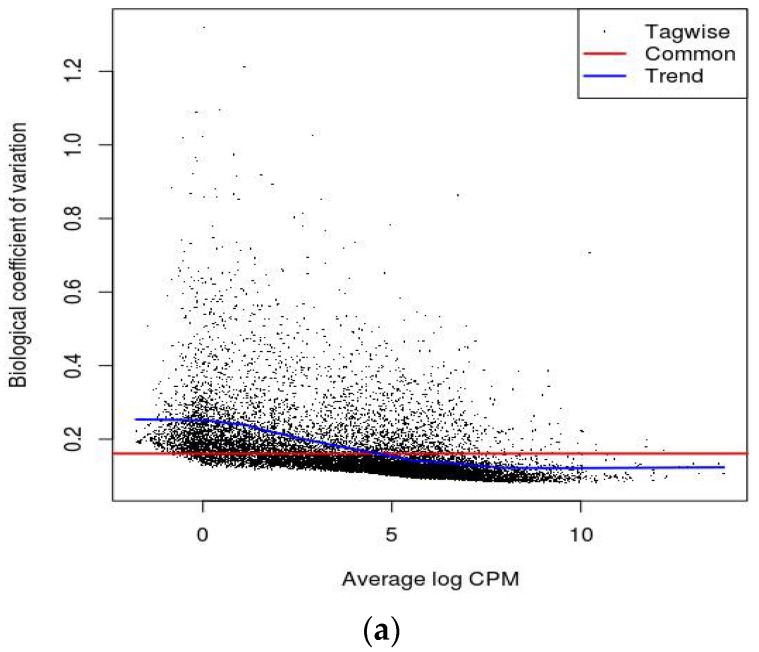

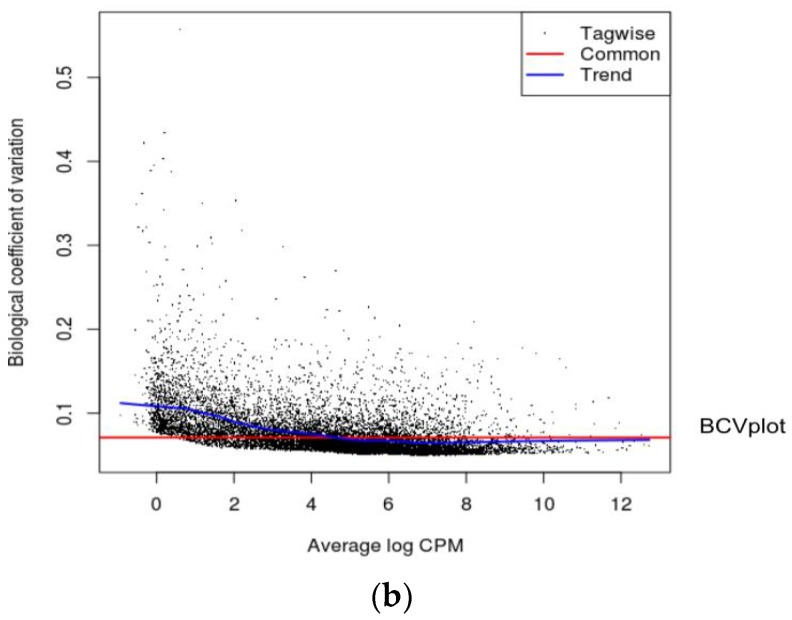
Biological Coefficient of Variation plots: (**a**) For JQ1 treatment; (**b**) For Dexamethasone treatment. CPM- counts per million.

**Figure 3:**
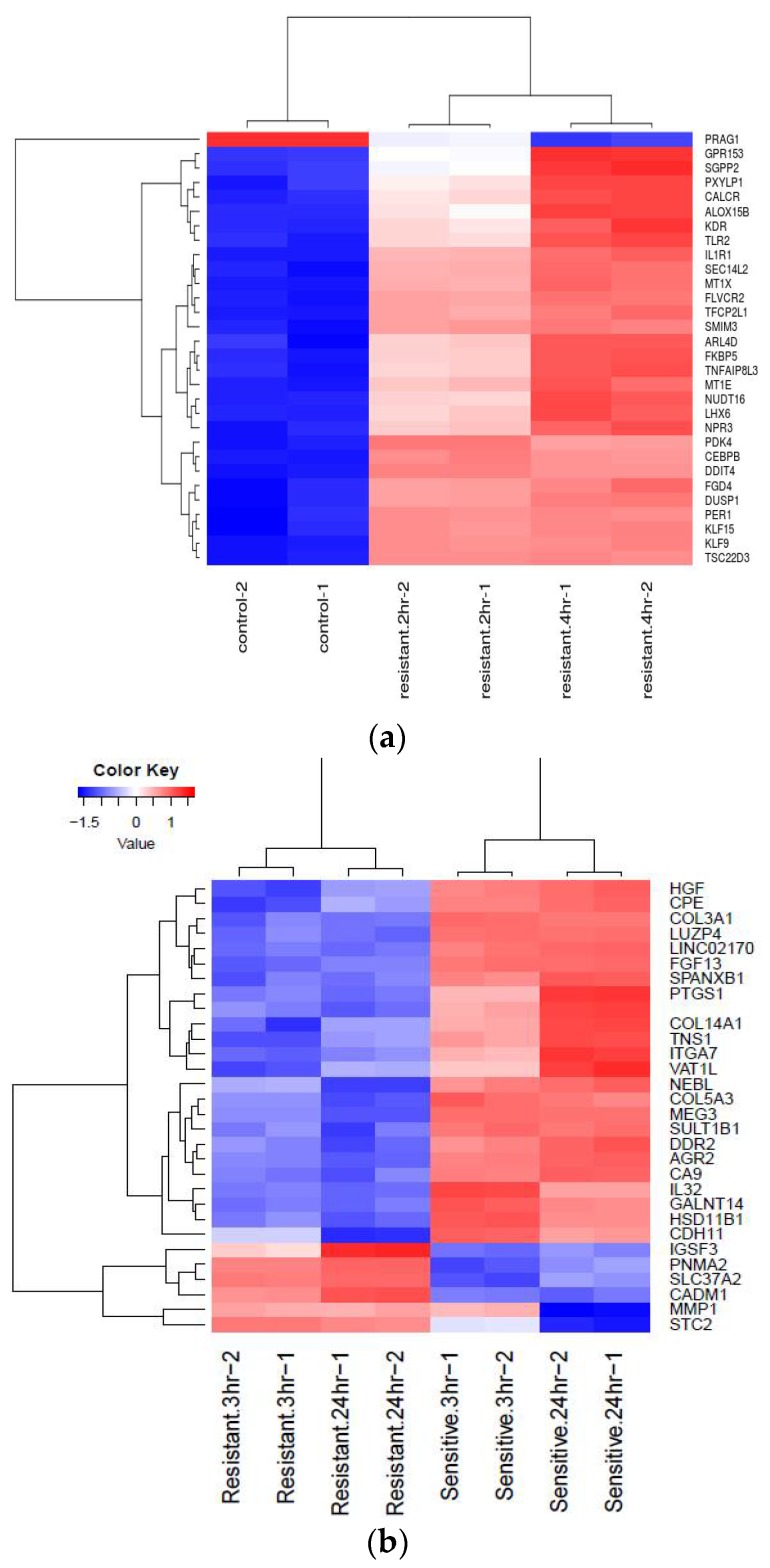
Heat maps: (**a**) For 24 h of JQ1 treatment; (**b**) For four hours of Dexamethasone treatment.

A multidimensional scaling (MDS) plot was generated which gives relative positions of each sample (Figure 1a,b). This plot helps in visually representing distances or dissimilarities between each group of datasets. The distance between the samples in the plot can be interpreted as the log fold change for the genes. The replicate samples cluster together while the samples from different groups are well separated [16]. Biological Coefficient of Variation (BCV) plots are used to visualize dispersion estimates obtained from the estimate Disp function in edgeR. A dispersion parameter is a representation of variability between biological replicates in a negative binomial distribution adopted in edgeR [16]. BCV or square-root dispersion was plotted on the y-axis of a BCV plot. It shows the coefficient of variation (Figure 2a,b). Heat map is a two-dimensional graphical representation of data where the individual values contained in a matrix are represented as colors. A matrix of significant DEGs are better represented in a heat map with upregulated and downregulated genes assigned with different colors [16]. This heat map gives the pattern of expressional changes of the top 30 significant differentially expressed genes (Figure 3a,b).

Functional annotation was done using gene ontology (GO) enrichment analysis for studying the biological roles of significant differentially expressed genes in the study. Broad classification of GO terms includes three groups: biological processes (BP), cellular components (CC), and molecular function (MF). The most significantly enriched processes found out in the study were multicellular organismal process, cell cycle, regulation of signal transduction, and response to stimulus. All of them belonged to biological processes (Table 3). The top GO terms for each sample are given in supplementary material (Supplementary Table S2a–d). Kyoto Encyclopedia of Genes and Genomes (KEGG) pathway analysis was done to identify the most significant pathways that had been altered in the samples of study. Cytokine-cytokine receptor interaction was the most significantly enriched pathway for three out of four different treatment samples taken in the study. Cell cycle was the other significantly enriched pathway (Table 4). The top ten pathways for each treatment sample are given in supplementary material (Supplementary Table S3a–d).

The importance of the cytokine-cytokine receptor interaction pathway has been highlighted by these studies and further investigation is required to target this pathway for therapeutic and diagnostic purposes.

**Figure 4:**
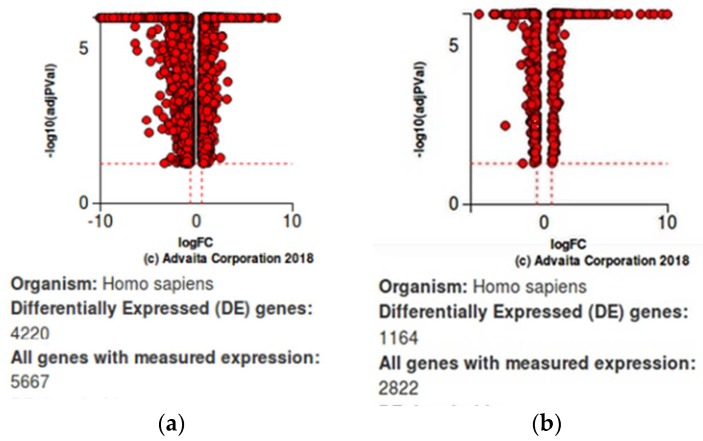
Volcano plots: (**a**) For 24 h of JQ1 treatment. DE thresholds: Fold change 0.6, *p*-value 0.05.; (**b**) For four hours of Dex treatment. x-axis: log fold change (log FC), y-axis: negative logarithm of adjusted *p*-value -log10 (adjPVal). DE thresholds: Fold change 0.6, *p*-value 0.05.

**Figure 5:**
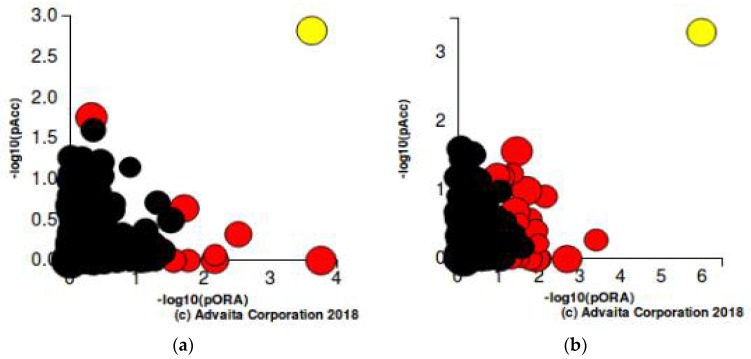
Perturbation (y-axis) vs. Overrepresentation (x-axis) reveals disrupted pathways: (**a**) At 24 h of JQ1 treatment; (**b**) At four hours of Dexamethasone treatment. The size of each dot denotes the number of genes in the pathway. The x-axis measures *p*-values obtained using the classical over-representation analysis (pORA). The y-axis represents the *p*-values obtained from total perturbation accumulation (pAcc) in the pathway. Yellow: Cytokine-cytokine receptor interaction pathway, Red: Significantly enriched pathways, Black: Non-significantly enriched pathways.

The iPathway Guide analysis was used for further validation of our findings of significance. This analysis revealed that the most significant altered pathway at all time intervals for both of the drug treatments was cytokine-cytokine receptor interaction with a *p*-value of 5.588 × 10^−6^ and 1.120 × 10^−8^ for JQ1 and dexamethasone, respectively. The PI3K-Akt signaling pathway was also found to be significantly altered. The list of top statistically significantly altered pathways is given in Table 5a,b.Volcano plots for differentially expressed genes (resistant vs. sensitive) are given in Figure 4a,b. Perturbation versus overrepresentation of disrupted pathways at 24 h of JQ1 treatment and four hours of Dex treatment are represented in Figure 5a,b.

## 3. Discussion

The aim of the present study was to explore changes at the molecular level in the drug-resistant TNBC cell lines, which included the study of differentially expressed genes, cellular processes, pathways, and pathway interactions. The difference seen in these molecular aspects of sensitive and resistant cells could throw light on the mechanism of resistance. The study could also give an insight into the major pathways and altered gene functions to be targeted for diagnostic and prognostic purposes in future. Among the different subtypes of TNBC, the study focused on Basal B subtypes and, therefore, two different cell lines (SUM159 and MDA-MB-231) belonging to the same subtype were used and checked for consistency as well as disparity between the two. The drugs used were JQ1 and Dexamethasone.

JQ1 is a bromodomain or extra terminal inhibitor or BETi and it stops cancer cells from adapting to hypoxia. Therefore, it acts as an important therapy agent in hard-to-treat breast cancers [17]. Dexamethasone is used as a pretreatment drug in chemotherapy to prevent severe side effects such as vomiting, nausea, and allergic reactions. They are used prior to, during and after chemotherapy at various doses to protect the normal tissues against the long-term effects of genotoxic drugs and to reduce toxicity. Studies have shown Dex to have an antiproliferative effect on certain types of breast cancer cell lines. Dex, when used as comedication during chemotherapy, was found to cause substantial immunomodulatory effects. Dex induces drug resistance in TNBC cells in some cases, in both in vivo and in vitro conditions as reported by Li Z et al. [12].

Only the significant factors common to both time intervals of each sample were considered. The upregulated genes with lowest *p*-values were *SLC37A2*, *PNMA2*, and *CADM1*. *SLC37A2* is a gene that codes for a protein that is involved in sugar-phosphate exchange [18]. A study has pointed to the reported role of solute carrier (SLC) proteins in drug resistance [19]. Therefore, further investigations have to be done to elucidate the role played by the *SLC37A2* gene in contributing to drug resistance. The significantly downregulated genes with the lowest *p*-values were *CDH11* and *HGF*. *CDH11*, also known as *OB Cadherin*, has a role in the metastasis process and has already been established as an epigenetic biomarker of drug resistance in acute myeloid leukemia. The class of proteins to which *CDH11* belongs has already been proposed as novel targets for anticancer therapy [20,21]. This is in the case of JQ1 treatment. In the case of Dexamethasone treatment, the top three upregulated genes with lowest *p*-values were *PDK4*, *TSC22D*, and *KLF9*. PDK4-Pyruvate Dehydrogenase Kinase 4 acts as a gatekeeper of the tricarboxylic acid (TCA) cycle by inactivating pyruvate dehydrogenase (PDH). *PDK4* expression is downregulated dramatically in most tumor types. *PDK4* is found to be a critical metabolic regulator of epithelial-mesenchymal transition (EMT) seen in acquired anticancer drug resistance [22]. The top three downregulated genes with lowest *p*-values were *ZNF703*, *SOX9*, and *PRAG1*. The *ZNF703* gene is a novel oncogene seen in a small percentage of breast cancers—the ones harboring 8p12 amplifications. Luminal breast cancer cell lines in which the *ZNF703* is overexpressed are seen to be resistant to tamoxifen through the activation of Akt/mTOR signaling [23].

Gene ontology (GO) analysis was performed for elucidation of biological roles of the differentially expressed genes. The most significantly enriched GO category for JQ1 at three hours was multicellular organismal processes which come under the category of biological processes, whereas at 24 h, it was cell cycle which again came under biological processes. In MDA-MB-231 cells treated with Dex at two hours, the most significantly enriched GO category was regulation of signal transduction, and at four hours it was response to stimulus; both belong under biological processes. KEGG pathway analysis was conducted in order to study the biological roles of the differentially expressed genes. The pathway with the lowest *p*-value for JQ1 treatment at three hours was cytokine-cytokine interaction, which came under the broader category of environmental information processing. At 24 h of JQ1 treatment, the most significantly enriched pathway was the cell cycle. The cytokine-cytokine interaction pathway was repetitively seen to be significantly enriched in both 2-h and 4-h treatment of MDA-MB-231 cells with Dex.

Cytokine signaling is said to direct tumor cell proliferation and stromal blood vessel network formation. There is evidence that cancer cells and their associated stroma secrete cytokines that play a key role in a number of drug-resistant mechanisms [24]. Cancer stromal cells secrete specific cytokines and these confer resistance to chemotherapy. Cytokines are seen to play a major role in cancer cell progression as well as the cancer drug resistance mechanism. It is essential to establish a correlation between the cytokine profile and cancer drug resistance. Exosomes, which are secreted by various types of cells in the microenvironment of the tumor, transfer bioactive molecules such as micro RNAs and cytokines that are seen to play an essential role in tumor progression and therapy resistance in the tumor cells [25]. The gene ontology (GO) enrichment analysis and the KEGG pathway enrichment analysis are the common downstream procedures to interpret the differential expression results in a biological context [26]. Given a set of genes that are up- or downregulated under a certain contrast of interest, a GO (or pathway) enrichment analysis will find which GO terms (or pathways) are over- or underrepresented using annotations for the genes in that set.

Crucial information, like the type of each gene, the position of each gene on each pathway, and the type and directions of the interactions with the other genes is essential to carry out a more meaningful pathway analysis.

iPathway Guide is an implementation of a pathway analysis approach [27] introduced a few years ago. This technique uses a systems biology approach that takes into consideration the direction and type of every edge on every pathway, the location of every gene, etc. This has been shown to both eliminate many of the false positives produced by the other approaches, as well as correctly identify true positives that are otherwise missed. Our analysis with iPathway Guide cross-validated our findings of the significance of cytokine-cytokine receptor interactions in the present study.

The limitation of the present study was the unavailability of datasets of similar nature viz. RNASeq data of TNBC resistant samples. More studies on the transcriptome drug-resistant samples in TNBC would have provided greater heterogeneity in the sample subsets, and hence given a broader perspective on the molecular aspects. The results of this study give the molecular characteristics of the drug-resistant cell lines as compared to drug-sensitive, and these can be considered for determining therapeutic targets. Further studies on the cytokine-cytokine interaction pathway and its components should be carried out for arriving at further conclusions regarding overcoming of drug resistance for successful treatment of TNBC.

In conclusion, the present study performed an analysis of the RNAseq datasets available of drug-resistant TNBC cell lines to identify the top differentially expressed genes, pathways, as well as other biological functions. The cytokine-cytokine interaction pathway showed a consistent significant differential expression in both the different cell lines. With evidence from other published literature and our findings, we were able to conclude that this pathway could be targeted for therapy. Further functional studies can give an insight into the role of the significantly expressed biological factors in the resistant samples to throw light on the mechanism of resistance and how to overcome the same using targeted therapies. Sun X. et al. confirmed that crosstalk inhibition diminishes the response of signaling output to the external stimuli [28]. Therefore, further studies are required to confirm the dependability on present findings to be adopted as therapeutic targets.

## 4. Materials and Methods

Gene expression profiling studies related to drug resistance in TNBC were identified by searching the Gene Expression Omnibus (GEO) database [29]. The following combination of key words were used: “triple-negative breast cancer”, “drug resistance” and “RNASeq”. RNASeq data of cell lines exhibiting drug-resistant nature (SUM 159R and MDA-MB231) and the corresponding parental/control cell lines were used to carry out a differential expression analysis at the provided time intervals. Two experimental RNASeq datasets that dealt with drug resistance in TNBC cell lines were downloaded from the GEO database (Table 6).

The characteristics of the experimental setups that generated the datasets were as follows:

(1) The first study [30] was based on the effect of bromodomain inhibitors on SUM159 cell lines. In the RNASeq experiment, SUM159 (drug-sensitive) and SUM159R (drug-resistant) were incubated in biological duplicates for 3, 12 and 24 h with 500 nM of JQ1 or Dimethyl sulfoxide (DMSO) treatment. For our study, available duplicate reads (GSE63582) at both 3 and 24 h of JQ1 treatment on SUM159 and SUM159R were downloaded from the database.
(2) The second experimental study [31] focused on establishing the relationship between dexamethasone (a coadjuvant) and drug resistance in MDA-MB-231 cell lines. MDA-MB-231 cells were treated with 100 nM Dex or 10 μM CpdA for two and four  hours, respectively. For our study, all duplicate reads (GSE56022) for control, after two and after four hours of drug treatment, were downloaded.

RNASeq data are usually very large and require efficient algorithms which use minimum computing resources for their analysis [19]. The major steps in an RNASeq analysis pipeline include alignment of sequence reads to a reference genome, quantification of transcripts, and differential expression, which output a list of up- and downregulated genes.

The RNASeq reads were assessed for their quality using the Fast QC 0.11.6 [32] program and aligned to GRCh38 reference genome using STAR-2.5.3a [33] algorithm. The transcripts were quantified using subread-1.6.0 [34] and differentially expressed genes were determined using the edgeR v3.22.2 [35] program. GO enrichment and KEGG pathway enrichment were performed. The details of program codes are in the supplementary data. The lists of differentially expressed genes from edgeR were further analyzed using iPathway Guide from Advaita Bioinformatics [28].

Top differentially expressed genes were identified in both of the experimental datasets (SUM159R vs. SUM159 and MDA-MB-231 vs. control) at different time intervals of drug treatment. Drug-sensitive samples corresponding to each treatment were taken as controls.

The edgeR program in R studio was used to determine how the genes are differentially expressed in the drug-resistant cells when compared to the sensitive ones. edgeR uses probabilistic methods for determining differential expression. The genes and pathways affected were determined based on a false discovery rate (FDR) of 0.05 and a log FC of 1 [16]. edgeR filters genes with low transcript count after which normalization of all the samples was done.

## Figures and Tables

**Table 1:** Total number of differentially expressed genes (DEGs) at each time interval of drug treatment.

Drug Treatment	Time Interval	Cell Lines Contrast	Total No. of Significant DEGs	No. of Upregulated Genes	No. of Downregulated Genes
JQ1	3 h	SUM159R vs. SUM159	3580	1310	2270
JQ1	24 h	SUM159R vs. SUM159	2682	891	1791
Dexamethasone	2 h	MDA-MB-231 vs. control	301	204	97
Dexamethasone	4 h	MDA-MB-231 vs. control	563	325	238

**Table 2:** Top most significantly up-& downregulated differentially expressed genes.

Regulation	Entrezid	Gene Symbol	Log FC	*p*-value	FDR
Up-regulated					
JQ1.3 h	219,855	*SLC37A2*	4.6143434	2.621552 × 10^−164^	3.786831 × 10^−160^
JQ1.24 h	23,705	*CADM1*	4.3898554	7.165931 × 10^−122^	2.070238 × 10^−118^
Dex.2 h	5166	*PDK4*	8.6764105	<1 × 10^−3^	<1 × 10^−3^
Dex.4 h	5166	*PDK4*	7.8302047	<1 × 10^−3^	<1 × 10^−3^
Down-regulated					
JQ1.3 h	1634	*DCN*	−4.8813506	1.516128 × 10^−153^	1.095024 × 10^−149^
JQ1.24 h	1009	*CDH11*	−7.3956023	1.073032 × 10^−160^	1.549994 × 10^−15^
Dex.2 h	10,769	*PLK2*	−2.5506627	5.018664 × 10^−97^	2.665914 × 10^−94^
Dex.4 h	157,285	*PRAG1*	−2.6394177	1.108977 × 10^−207^	1.132863 × 10^−204^

Dex—Dexamethasone; FDR—False Discovery Rate.

**Table 3:** Most significantly enriched Gene Ontology (GO) terms for different samples.

Treatment	GO term	N	Up	Down	*p*-Value(Up)	*p*-Value (Down)
JQ1.3 h	Multicellular organismal process	4185	884	1539	0.9999998	2.897229 × 10^−46^
JQ1.24 h	Cell cycle	1550	552	173	8.399527 × 10^−38^	1.000000 × 10
Dex.2 h	Regulation of signal transduction	2053	170	198	3.582583 × 10^−14^	2.810198 × 10^−22^
Dex.4 h	Response to stimulus	5230	652	776	1.06389 × 10^−19^	1.297062 × 10^−7^

**Table 4:** Most significantly enriched Kyoto Encyclopedia of Genes & Genomes (KEGG) pathways for different samples.

Treatment	KEGG Pathway	N	Up	Down	*p*-Value (Up)	*p*-Value (Down)
JQ1.3 h	Cytokine-cytokine receptor interaction	113	16	64	9.962870 × 10^−1^	3.082753 × 10^−10^
JQ1.24 h	Cell cycle	117	57	5	2.416778 × 10^−10^	1.000000 × 10
Dex.2 h	Cytokine-cytokine receptor interaction	109	10	31	3.648702 × 10^−2^	9.283461 × 10^−16^
Dex.4 h	Cytokine-cytokine receptor interaction	109	12	44	3.591688 × 10^−1^	7.440649 × 10^−13^

**Table 5:** Statistically significant altered pathways (Resistant vs. Sensitive).

**(a) 24 h of JQ1 Treatment**	
**Pathway**	***p*-Value**
Cytokine-cytokine receptor interaction	5.588 × 10^−6^
Neuroactive ligand-receptor interaction *	1.708 × 10^−4^
Cell adhesion molecules (CAMs) *	0.007
Systemic lupus erythematosus	0.011
Drug metabolism-cytochrome P450 *	0.017
Metabolism of xenobiotics by cytochrome P450 *	0.028
Chemical carcinogenesis *	0.028
Rap1 signaling pathway	0.028
*Staphylococcus aureus* infection	0.035
PI3K-Akt signaling pathway	0.048
**(b) 4 h of Dexamethasone Treatment**	
**Pathway**	***p*-Value**
Cytokine-cytokine receptor interaction	1.120 × 10^−8^
Rheumatoid arthritis	0.002
Neuroactive ligand-receptor interaction *	0.002
AGE-RAGE signaling pathway in diabetic complications	0.007
Pathways in cancer	0.008
Hematopoietic cell lineage *	0.010
Transcriptional misregulation in cancer *	0.012
PI3K-Akt signaling pathway	0.015
Phosphatidylinositol signaling system *	0.016
Basal cell carcinoma	0.018

* overrepresentation only. AGE-RAGE: Advanced glycation end products–Receptor for advanced glycation end products. Rap1: Ras-proximate-1.

**Table 6:** RNASeq datasets used for the study and their characteristics.

GEO ID	Sequencing Platform	Cell Lines	Time Interval	Treatment
GSE63582	Illumina True-Seq	SUM 159 (sensitive)	3 h	JQ1 (500 nM)
		SUM 159 (sensitive)	24 h	
		SUM 159R	3 h	
		SUM 159R	24 h	
GSE56022	Illumina Hi-Seq	MDA-MB 231	2 h	Dexamethasone (100 nM)
		MDA-MB 231	4 h	
		MDA-MB 231 (control 1)	2 h	
		MDA-MB 231 (control 2)	4 h	

GEO—Gene Expression Omnibus.

## Supplementary Materials

Supplementary materials can be found at http://www.mdpi.com/1422-0067/19/6/1810/s1.

## Abbreviations

TNBC	Triple-Negative Breast Cancer
ER	Estrogen Receptor
PR	Progesterone receptor
HER2	Human Epidermal Growth Factor Receptor 2
DEGs	Differentially Expressed Genes
GO	Gene Ontology
KEGG	Kyoto Encyclopedia of Genes and Genomes
BL	Basal-Like
PARP-1	poly(ADP-ribose) polymerase-1
EGFR	epidermal growth factor receptor
FGFR2	fibroblast growth factor receptor 2
VEGF	vascular endothelial growth factor
mTOR	the mammalian target of rapamycin
AMPK	5′ adenosine monophosphate-activated protein kinase
PI3K	phosphatidylinositol-3-kinase
NGS	Next-Generation Sequencing
Dex	Dexamethasone
Log FC	log Fold Change
FDR	False Discovery Rate
BCV	Biological Coefficient of Variation
CPM	Counts per Million
AGE-RAG	Advanced glycation end products–Receptor for advanced glycation end products
Rap-1	Ras-proximate-1
SLC	solute carrier
BP	Biological Processes
CC	Cellular Components
MF	Molecular Function
TCA	Tricarboxylic acid cycle
DMSO	Dimethyl Sulphoxide
GEO	Gene Expression Omnibus
