# Supplementary material for: Differential Expression and Pathway Analysis in Drug-Resistant Triple-Negative Breast Cancer Cell Lines Using RNASeq Analysis
Source: Int J Mol Sci. 2018 Jun 19;19(6):1810. doi: 10.3390/ijms19061810 (PMC6032108; doi:10.3390/ijms19061810)
Supplement: Supplementary file 1 [file ijms-19-01810-s001.docx]

**SUPPLEMENTARY TABLES**

**TABLES 1a-1d. Top 10 significant differentially expressed genes(DEGs)**

1a. JQ1.3hr

| ENTREZID SYMBOL logFC PValue FDR |
| --- |
| 219855 SLC37A2 4.6143434 2.621552e^-164^ 3.786831e^-160^  1634 DCN -4.8813506 1.516128e^-153^ 1.095024e^-149^  3371 TNC -4.5958354 8.300313e^-152^ 3.996601e^-148^  3638 INSIG1 -3.5065667 4.005842e^-128^ 1.446610e^-124^  55384 MEG3 -5.2139641 9.482569e^-125^ 2.739514e^-121^  1009 CDH11 -4.8122883 4.076645e^-122^ 9.814522e^-119^  9235 IL32 -5.3758888 1.275808e^-121^ 2.632721e^-118^  3082 HGF -4.9302847 1.358685e^-117^ 2.453275e^-114^  4940 OAS3 -4.6452433 2.354524e^-117^ 3.779011e^-114^  834 CASP1 -4.5716366 8.913301e^-117^ 1.287526e^-113^ |

1b. JQ1.24hr

| ENTREZID SYMBOL logFC PValue FDR |
| --- |
| 1009 CDH11 -7.3956023 1.073032e^-160^ 1.549994e^-156^  55384 MEG3 -6.3018920 2.623890e^-152^ 1.895104e^-148^  5742 PTGS1 -4.2225546 5.901235e^-128^ 2.131083e^-124^  23705 CADM1 4.3898554 7.165931e^-122^ 2.070238e^-118^  8614 STC2 3.4740592 4.246083e^-119^ 1.022245e^-115^  219855 SLC37A2 3.7116638 1.511649e^-117^ 3.119395e^-114^  3679 ITGA7 -5.8155837 1.060458e^-115^ 1.914789e^-112^  768 CA9 -8.3276458 6.554078e^-115^ 1.051930e^-111^  27284 SULT1B1 -8.8866256 1.940065e^-113^ 2.802424e^-110^ |

1c. Dex.2hr

| ENTREZID SYMBOL logFC PValue FDR |
| --- |
| 5166 PDK4 8.6764105 0.000000e^+00^ 0.000000e^+00^  1831 TSC22D3 5.1050496 0.000000e^+00^ 0.000000e^+00^  687 KLF9 3.9449548 3.228586e^-258^ 1.429188e^-254^  1051 CEBPB 3.1462051 2.349587e^-251^ 7.800629e^-248^  54541 DDIT4 2.7905177 2.434831e^-246^ 6.466912e^-243^  2289 FKBP5 3.5297690 1.882829e^-224^ 4.167328e^-221^  85027 SMIM3 3.7190200 8.545947e^-207^ 1.621288e^-203^  55640 FLVCR2 3.2532699 3.338876e^-204^ 5.542535e^-201^  29842 TFCP2L1 3.1845020 8.107758e^-169^ 1.196345e^-165^  1843 DUSP1 2.9658795 1.684071e^-144^ 2.236446e^-141^ |

1d. Dex.4hr

| ENTREZID SYMBOL logFC PValue FDR |
| --- |
| 5166 PDK4 7.8302047 0.000000e^+00^  0.000000e^+00^  1831 TSC22D3 5.1632590 0.000000e^+00^  0.000000e^+00^  2289 FKBP5 5.0379933 0.000000e^+00^  0.000000e^+00^  687 KLF9 4.0685781 1.731787e^-273^ 5.749532e^-270^  55640 FLVCR2 3.7186863 3.987454e^-267^  1.059068e^-263^  85027 SMIM3 4.0579227 1.217605e^-239^  2.694966e^-236^  1051 CEBPB 2.9954752 1.466172e^-230^  2.781537e^-227^  54541 DDIT4 2.6681368 6.693412e^-227^  1.111106e^-223^  29842 TFCP2L1 3.6968149 1.131181e^-219^ 1.669120e^-216^  3554 IL1R1 2.9359980 1.352880e^-214^  1.796624e^-211^ |

**TABLES 2a-2d. Top 10 significantly enriched GO terms**

**2a. GO.JQ1.3hr**

| **GO ID** | **GO Term** | **Ont N Up Down** | **P.Up** | **P.Down** |
| --- | --- | --- | --- | --- |
| GO:0032501  GO:0005886  GO:0023052  GO:0071944  GO:0007275  GO:0048856  GO:0048731  GO:0051239  GO:0007154  GO:0050896 | multicellular organismal process  plasma membrane  signaling  cell periphery  multicellular organism development  anatomical structure development  system development  regulation of multicellularorganismal process  cell communication  response to stimulus | BP 4185 884 1539  CC 2747 590 1035  BP 3859 803 1379  CC 2823 606 1058  BP 3221 670 1182  BP 3540 738 1278  BP 2869 581 1069  BP 1813 350 728  BP 3876 808 1377  BP 5439 1184 1840 | 0.9999998  0.9995913  1.0000000  0.9996878  0.9999990  0.9999997  0.9999999  0.9999998  0.9999999  0.9999991 | 2.897229e^-46^  2.836443e^-32^  4.189443e^-32^  4.914754e^-32^  6.638825e^-32^  2.363920e^-31^  3.146917e^-31^  4.997296e^-31^  1.352076e^-30^  2.499053e^-30^ |
| Gene Ontology, GO | | | | |

**2b. GO.JQ1.24hr**

| **GO ID** | **GO Term** | **Ont N Up Down** | **P.Up** | **P.Down** |
| --- | --- | --- | --- | --- |
| GO:0007049  GO:0022402  GO:0005694  GO:0043232  GO:0043228  GO:0044428  GO:0000278  GO:0031981  GO:1903047  GO:0006260 | cell cycle  cell cycle process  chromosome  intracellular non-membrane-bounded organelle  non-membrane-bounded organelle  nuclear part  mitotic cell cycle  nuclear lumen  mitotic cell cycle process  DNA replication | BP 1550 552 173  BP 1124 418 119  CC 831 331 89  CC 3209 956 479  CC 3209 956 479  CC 3694 1070 473  BP 892 342 90  CC 3417 1000 433  BP 755 301 72  BP 264 141 19 | 8.399527e^-38^  7.927658e^-33^  3.063775e^-32^  5.516554e^-31^  5.516554e^-31^  4.508714e^-30^  1.461913e^-29^  2.094484e^-29^  2.290080e^-29^  8.170572e^-29^ | 1.000000e^+00^  1.000000e^+00^  1.000000e^+00^  1.000000e^+00^  1.000000e^+00^  1.000000e^+00^  1.000000e^+00^  1.000000e^+00^  1.000000e^+00^  1.000000e^+00^ |

**2c. GO.dex.2hr**

| **GO ID** | **Term** | **Ont N Up Down** | **P.Up** | **P.Down** |
| --- | --- | --- | --- | --- |
| GO:0009966 | regulation of signal transduction | BP 2053 170 198 | 3.582583e^-14^ | 2.810198e^-22^ |
| GO:0007165 | signal transduction | BP 3466 252 282 | 7.639391e^-15^ | 1.216323e^-21^ |
| GO:0048018 | receptor ligand activity | MF 143 10 42 | 1.487495e^-01^ | 1.542222e^-21^ |
| GO:0010646 | regulation of cell communication | BP 2206 179 205 | 3.748038e^-14^ | 3.639676e^-21^ |
| GO:0048583 | regulation of response to stimulus | BP 2567 188 226 | 1.005770e^-10^ | 1.333308e^-20^ |
| GO:0030545 | receptor regulator activity | MF 159 10 43 | 2.326474e^-01^ | 1.770380e^-20^ |
| GO:0023051 | regulation of signaling | BP 2235 181 205 | 3.010611e^-14^ | 1.822213e^-20^ |
| GO:0023052 | signaling | BP 3715 267 290 | 2.418664e^-15^ | 1.456969e^-19^ |
| GO:0007154 | cell communication | BP 3736 271 291 | 3.248509e^-16^ | 1.662405e^-19^ |
| GO:0048518 | positive regulation of biological process | BP 3743 257 291 | 3.989144e^-12^ | 2.238179e^-19^ |

**2d. GO.dex.4hr**

| **GO ID** | **Term** | **Ont N Up Down** | **P.Up** | **P.Down** |
| --- | --- | --- | --- | --- |
| GO:0050896 | response to stimulus | BP 5230 652 776 | 1.063389e^-19^ | 1.297062e^-07^ |
| GO:0007154 | cell communication | BP 3736 498 588 | 3.490723e^-19^ | 2.551061e^-09^ |
| GO:0023051 | regulation of signaling | BP 2235 329 397 | 8.128534e^-18^ | 5.564429e^-13^ |
| GO:0007165 | signal transduction | BP 3466 463 561 | 1.337879e^-17^ | 6.555782e^-11^ |
| GO:0023052 | signaling | BP 3715 489 587 | 1.518813e^-17^ | 1.340443e^-09^ |
| GO:0009966 | regulation of signal transduction | BP 2053 307 371 | 1.697840e^-17^ | 3.628695e^-13^ |
| GO:0010646 | regulation of cell communication | BP 2206 324 394 | 2.332028e^-17^ | 3.047508e^-13^ |
| GO:0051716 | cellular response to stimulus | BP 4415 555 688 | 3.774391e^-16^ | 1.821614e^-10^ |
| GO:0009605 | response to external stimulus | BP 1232 201 225 | 5.019786e^-15^ | 1.813005e^-08^ |

**TABLES 3a-3d. Top 10 significantly enriched KEGG pathways**

**3a. KEGG JQ1.3hr**

| **KEGG ID** | **Pathway** | **N Up Down** | **P.Up** | **P.Down** |
| --- | --- | --- | --- | --- |
| path:hsa04630  path:hsa00190  path:hsa05150  path:hsa05412  path:hsa05164  path:hsa05012  path:hsa05146  path:hsa05414  path:hsa04064  path:hsa05165 | Cytokine-cytokine receptor interaction  Ribosome  ECM-receptor interaction  Neuroactive ligand-receptor interaction  Protein digestion and absorption  Hematopoietic cell lineage  Cell adhesion molecules (CAMs)  Calcium signaling pathway  PI3K-Akt signaling pathway  Focal adhesion | 113 16 64  128 63 4  56 5 36  70 10 40  44 4 28  45 10 28  69 12 38  94 14 47  236 42 98  154 20 69 | 9.962870e^-01^  3.483575e^-10^  9.989517e^-01^  9.832236e^-01^  9.968604e^-01^  6.584285e^-01^  9.245183e^-01^  9.883617e^-01^  9.905642e^-01^  9.997609e^-01^ | 3.082753e^-10^  1.000000e^+00^  2.620335e^-08^  4.690348e^-07^  1.270122e^-06^  2.449419e^-06^  3.113188e^-06^  8.082182e^-06^  1.007360e^-05^  1.053615e^-05^ |

**3b. KEGG.JQ1.24hr**

| **KEGG ID** | Pathway | N Up Down | P.Up | P.Down |
| --- | --- | --- | --- | --- |
| path:hsa05322  path:hsa04110  path:hsa03030  path:hsa04514  path:hsa03013  path:hsa03008  path:hsa00982  path:hsa04060  path:hsa05034  path:hsa04974 | Systemic lupus erythematosus  Cell cycle  DNA replication  Cell adhesion molecules (CAMs)  RNA transport  Ribosome biogenesis in eukaryotes  Drug metabolism - cytochrome P450  Cytokine-cytokine receptor interaction  Alcoholism  Protein digestion and absorption | 54 7 34  117 57 5  35 24 1  69 15 34  144 59 11  74 36 2  32 2 19  113 26 45  103 17 42  44 8 23 | 9.715139e^-01^  2.416778e^-10^  6.230280e^-09^  5.891530e^-01^  3.292563e^-07^  5.029296e^-07^  9.968169e^-01^  4.613287e^-01^    9.412457e^-01^  7.949068e^-01^ | 2.932078e^-11^  1.000000e^+00^  9.997480e^-01^  1.749260e^-07^  9.999972e^-01^  9.999995e^-01^  2.602203e^-06^  4.083420e^-06^  4.158355e^-06^  4.764996e^-06^ |

**3c. KEGG.dex.2hr**

| **KEGG ID** | **Pathway** | **N Up Down** | **P.Up** | **P.Down** |
| --- | --- | --- | --- | --- |
| path:hsa04060 | Cytokine-cytokine receptor interaction | 109 10 31 | 3.648702e^-02^ | 9.283461e^-16^ |
| path:hsa04668 | TNF signaling pathway | 92 9 23 | 3.225330e^-02^ | 9.022691e^-11^ |
| path:hsa05200 | Pathways in cancer | 366 30 44 | 2.995618e^-03^ | 5.734875e^-08^ |
| path:hsa04390 | Hippo signaling pathway | 118 9 20 | 1.139538e^-01^ | 1.476989e^-06^ |
| path:hsa05224 | Breast cancer | 98 6 18 | 3.309759e^-01^ | 1.484101e^-06^ |
| path:hsa04010 | MAPK signaling pathway | 213 15 27 | 8.873922e^-02^ | 8.153170e^-06^ |
| path:hsa04657 | IL-17 signaling pathway | 62 5 13 | 1.761561e^-01^ | 9.486179e^-06^ |
| path:hsa05226 | Gastric cancer | 102 6 17 | 3.654889e^-01^ | 1.132432e^-05^ |
| path:hsa04064 | NF-kappa B signaling pathway | 67 13 13 | 1.466688e^-05^ | 2.299835e^-05^ |
| path:hsa04310 | Wnt signaling pathway | 97 2 16 | 9.507868e^-01^ | 2.333761e^-05^ |

**3d. KEGG.dex.4hr**

| KEGG ID | Pathway | N Up Down | P.Up | P.Down |
| --- | --- | --- | --- | --- |
| path:hsa04060 | Cytokine-cytokine receptor interaction | 109 12 44 | 3.591688e^-01^ | 7.440649e^-13^ |
| path:hsa04668 | TNF signaling pathway | 92 12 32 | 1.735450e^-01^ | 7.040762e^-08^ |
| path:hsa05200 | Pathways in cancer | 366 60 81 | 2.740719e^-05^ | 6.982075e^-07^ |
| path:hsa05213 | Endometrial cancer | 55 17 6 | 9.433203e^-06^ | 7.370581e^-01^ |
| path:hsa04151 | PI3K-Akt signaling pathway | 222 42 41 | 1.489945e^-05^ | 1.213558e^-02^ |
| path:hsa05206 | MicroRNAs in cancer | 131 23 35 | 3.328962e^-03^ | 1.842835e^-05^ |
| path:hsa05226 | Gastric cancer | 102 19 29 | 3.724508e^-03^ | 2.688035e^-05^ |
| path:hsa05224 | Breast cancer | 98 20 28 | 9.375292e^-04^ | 3.333762e^-05^ |
| path:hsa05216 | Thyroid cancer | 34 12 4 | 4.478983e^-05^ | 6.616691e^-01^ |
| path:hsa04064 | NF-kappa B signaling pathway | 67 14 21 | 4.167655e^-03^ | 7.239983e^-05^ |

**SUPPLEMENTARY DATA- COMMAND LINES AND CODES FOR ALL THE STEPS IN PROGRAM**

The RNA-Seq analysis pipeline consisted of the following steps and programs. The same steps were followed for both of the experimental data sets.

1. ***Download of the RNA-Seq data from SRA****(a single sra file selected for illustration)*.

$wget [ftp://ftp-trace.ncbi.nih.gov/sra/sra-instant/reads/ByRun/sra/SRR/SRR166/SRR1661334/SRR1661334.sra](ftp://ftp-trace.ncbi.nih.gov/sra/sra-instant/reads/ByRun/sra/SRR/SRRid/sampleid/sampleid.sra)

1. ***Conversion of sra files to fastq files using sra tool kit***( <https://ncbi.github.io/sra-tools/install_config.html> )

$ ./fastq-dump /path/to/ SRR1661334.sra

1. ***Quality check of fastq files using FastQC program*** ( <http://www.bioinformatics.babraham.ac.uk/projects/fastqc/>).

$ ./fastqc -o /path/to/output directory/ /path/to/R1.fastq

The FastQC reports of all the samples were analysed and found that all the reads had an average Phred quality score of above 30 (99.9% accuracy).

1. ***Alignment of sample reads to GRCh38 reference genome using STAR algorithm*** ( <https://github.com/alexdobin/STAR> ) .

Before aligning the reads the reference genome (fasta) and annotation (gtf) files needs to be downloaded and reference genome needs to be indexed :

$ wget <ftp://ftp.ensembl.org/pub/release-90/fasta/homo_sapiens/dna/Homo_sapiens.GRCh38.dna.primary_assembly.fa.gz>

$ gunzip Homo_sapiens.GRCh38.dna.primary_assembly.fa.gz

$ wget <ftp://ftp.ensembl.org/pub/release-90/gtf/homo_sapiens/Homo_sapiens.GRCh38.90.gtf.gz>

$ gunzip Homo_sapiens.GRCh38.90.gtf.gz

A directory named 'genome' is created under STAR directory and command for indexing is run in that directory.

$ ~/STAR-2.5.3a/bin/Linux_x86_64/STAR --runThreadN 8 --runMode genomeGenerate --genomeDir ~/STAR-2.5.3a/genome/ --genomeFastaFiles ./Homo_sapiens.GRCh38.dna.primary_assembly.fa --sjdbGTFfile ~/STAR-2.5.3a/Homo_sapiens.GRCh38.90.gtf --sjdbOverhang 49

The indexed files are in the 'genome directory'.

The command for alignment goes as follows:

$ ~/STAR-2.5.3a/bin/Linux_x86_64/STAR --runThreadN 8 --genomeDir ~/STAR-2.5.3a/genome/ --sjdbGTFfile ~/STAR-2.5.3a/Homo_sapiens.GRCh38.90.gtf --sjdbOverhang 49 --readFilesIn /path /to/R1.fastq

The output file is in SAM (Sequence Alignment Map) format.

An average unique mapping percentage of 81 % was obtained after aligning all sample reads.

1. ***Conversion of sam files to bam files***:

The sam files were converted to its binary version ( BAM format) using samtools( <http://samtools.sourceforge.net/>):

$ ./samtools view -b -S -o /path/to/R1.bam /path/to/R1.sam

6.***Quality check of bam files using RSeQC*** ( <http://rseqc.sourceforge.net/> ).

$ bam_stat.py -i /path/to/R1.bam

There were no reads that failed QC and there were no unmapped reads.

7. ***Quantification of transcripts using Subread*** ( <https://sourceforge.net/projects/subread/> ):

All the bam files corresponding to control and treated sample reads for each experiment /drug were fed to featurecounts function of subread.

$ featureCounts -t exon -g geneid -a /path/to/ Homo_sapiens.GRCh38.90.gtf.gz -o counts.txt /path/to/R1.bam /path/to/R2.bam /path/to/R3.bam /path/to/R4.bam /path/to/S1.bam /path/to/S2.bam /path/to/S3.bam/ /path/to/S4.bam

The output, 'counts.txt' file has the transcript counts corresponding to all genes for each sample read.

8.***Differential Expression using edgeR***

The 'counts' file is fed into edgeR (<https://bioconductor.org/packages/release/bioc/html/edgeR.html> ) program in R studio to determine how the genes are differentially expressed in the drug resistant cells when compared to the sensitive ones. edgeR uses probabilistic methods for determining differential expression. The genes and pathwaysaffected were determined based on an FDR of 0.05 and a logFC of 1.

The R code for estimating differential expression of genes and pathway analysis for each drug (eg. JQ1 at 3hr) is as follows:

# *preparing gene count table*

>JQ1counts<- read.table(“ [\\path\\to\\JQ1counts.txt](file:///\\path\\to\\DMSOcounts.txt) “, header=TRUE, row.names=1)

>JQ1counts<- JQ1counts[ ,6:ncol(JQ1counts)]

>colnames(JQ1counts) <- c( “r1”,”r2”,”r3”,”r4”,”s1”,”s2”,”s3”,”s4”)

# *adding EntrezIDs*

>gnsJQ1 <- select(org.Hs.eg.db, row.names(JQ1counts), c("ENTREZID","SYMBOL"),"ENSEMBL")

>gnsJQ1<- gnsDMSO[!duplicated(gnsJQ1[,1]),]

# *creating DGElist object*

> target

Type Time

r1 Resistant 3hr

r2 Resistant 3hr

r3 Resistant 24hr

r4 Resistant 24hr

s1 Sensitive 3hr

s2 Sensitive 3hr

s3 Sensitive 24hr

s4 Sensitive 24hr

>Group <- factor(paste(target$Type, target$Time, sep = ”.”))

> cbind(target,group=Group)

Type Time Group

r1 Resistant 3hr Resistant.3hr

r2 Resistant 3hr Resistant.3hr

r3 Resistant 24hr Resistant.24hr

r4 Resistant 24hr Resistant.24hr

s1 Sensitive 3hr Sensitive.3hr

s2 Sensitive 3hr Sensitive.3hr

s3 Sensitive 24hr Sensitive.24hr

s4 Sensitive 24hr Sensitive.24hr

>y.JQ1<- DGEList(JQ1counts,genes = gnsJQ1,group = Group)

# *filtering and normalisation of counts*

> countsPerMillion.JQ1<- cpm(y.JQ1)

> countCheck.JQ1<- countsPerMillion.JQ1> 1

> keep <- which(rowSums(countCheck.JQ1) >= 2)

> y.JQ1<- y.JQ1[keep,]

> y.JQ1<- calcNormFactors(y.JQ1, method="TMM")

# *MDS plot*

> plotMDS(y.JQ1)

# *designing model matrix*

>design.JQ1<- model.matrix(~0+Group)

>colnames(design.JQ1) <- levels(Group)

# *estimating dispersion and BCV plot*

>y.JQ1<- estimateDisp(y.JQ1,design.JQ1)

**>**plotBCV(y.JQ1)

# *assigning contrasts*

>JQ1.contrasts= makeContrasts(ResistantvsSensitive.3hr=Resistant.3hr-Sensitive.3hr, ResistantvsSensitive.24hr=Resistant.24hr-Sensitive.24hr,levels = design.JQ1)

# *fit object and differential expression*

> fit.JQ1<- glmFit(y.JQ1 design.JQ1)

>lrt.JQ1.3hr <- glmLRT(fit.JQ1, contrast=JQ1.contrasts[,"ResistantvsSensitive.3hr"])

>JQ1_result.3hr = topTags(lrt.JQ1.3hr, n=Inf, adjust.method="BH", sort.by="PValue", p.value=0.05)

# *listing up-regulated genes*

> JQ1_result.3hr = as.dataframe(JQ1_result.3hr)

> topUp.JQ1.3hr = JQ1_result.3hr[ JQ1_result.3hr$logFC >0 ,]

# listing down-regulated genes

> topDown.JQ1.24hr = JQ1_result.3hr [JQ1_result$logFC < 0 , ]

# *construction of heatmap*

> countsPerMillion = cpm(y.JQ1 , prior.count=2, log = TRUE)

> rownames(countsPerMillion)= y.JQ1$genes$SYMBOL

> colnames(countsPerMillion)= paste(y.JQ1$samples$group,1:2,sep="-")

> o.JQ1.3hr = order(lrt.JQ1.3hr$table$PValue)

> countsPerMillion = countsPerMillion[o.JQ1.3hr[1:30],]

> countsPerMillion=t(scale(t(countsPerMillion)))

>heatmap.2(countsPerMillion,col=col.pan,Rowv= TRUE,scale= "none",trace="none",dendrogram = "both",cexRow=1,cexCol=1.4,density.info = "none",margins = c(10,9),lhei= c(2,10),lwid= c(2,6))

# *DE genes*

>deGenes.JQ1.3hr=decideTestsDGE(lrt.JQ1.3hr, adjust.method="BH", p.value=0.05, lfc=1)

>summary(deGenes.JQ1.3hr)

1*Resistant.3hr -1*Sensitive.3hr

Down 2270

NotSig 10865

Up 1310

> deGenes.JQ1.3hr=row.names(lrt.JQ1.3hr)[as.logical(deGenes.JQ1.3hr)]

>plotSmear(lrt.JQ1.3hr,de.tags = deGenes.JQ1.3hr)
